# The Effect of Coronary Artery Bypass Surgery on Interleukin-18 Concentration and Biomarkers Related to Vascular Endothelial Glycocalyx Degradation

**DOI:** 10.3390/ijms26125453

**Published:** 2025-06-06

**Authors:** Danijel Knežević, Lara Batičić, Božena Ćurko-Cofek, Tanja Batinac, Aleksandra Ljubačev, Lara Valenčić Seršić, Gordana Laškarin, Marko Zdravković, Maja Šoštarič, Vlatka Sotošek

**Affiliations:** 1 Department of Anesthesiology, Reanimatology, Emergency and Intensive Care Medicine, University of Rijeka, Braće Branchetta 20, 51000 Rijeka, Croatia; danijel.knezevic2@uniri.hr (D.K.); lara.valencic@uniri.hr (L.V.S.); vlatkast@uniri.hr (V.S.); 2 Department of Medical Chemistry, Biochemistry and Clinical Chemistry, Faculty of Medicine, University of Rijeka, Braće Branchetta 20, 51000 Rijeka, Croatia; lara.baticic@uniri.hr; 3 Department of Physiology, Immunology and Pathophysiology, Faculty of Medicine, University of Rijeka, Braće Branchetta 20, 51000 Rijeka, Croatia; gordana.laskarin@uniri.hr; 4 Department of Clinical Medical Sciences I and II, Faculty of Health Studies, University of Rijeka, Viktora Cara Emina 2, 51000 Rijeka, Croatia; 5 Department of Surgery, Faculty of Medicine, University of Rijeka, Braće Branchetta 20, 51000 Rijeka, Croatia; aleksandra.ljubacev@uniri.hr; 6 Hospital for Medical Rehabilitation of Hearth and Lung Diseases and Rheumatism “Thalassotherapia-Opatija”, M. Tita 188, 51410 Opatija, Croatia; 7 Department of Anaesthesiology, Intensive Care and Pain Management, University Medical Centre Maribor, Ljubljanska ulica 5, 2000 Maribor, Slovenia; markozdravkovic@gmail.com; 8 Clinical Department of Anaesthesiology and Surgical Intensive Care, University Medical Centre, Zaloska 7, 1000 Ljubljana, Slovenia; maja.sostaric@kclj.si; 9 Department of Anesthesiology and Reanimatology, Faculty of Medicine, University of Ljubljana, Vrazov trg 2, 1000 Ljubljana, Slovenia

**Keywords:** cardiac surgery, endothelial glycocalyx, hyaluronic acid, interleukin-18, inflammation, syndecan-1

## Abstract

Surgical myocardial revascularization, regardless of the technique used, causes ischemia–reperfusion injury (IRI) in the myocardium mediated by inflammation and degradation of the endothelial glycocalyx (EG). We investigated the difference between on-pump and off-pump techniques in terms of the concentration of proinflammatory interleukin (IL)-18 and the EG degradation products syndecan-1 and hyaluronic acid measured by ELISA in the peripheral and cardiac circulation during open heart surgery and in the early postoperative period. The concentration of IL-18, C-reactive protein (CRP), and cardiac troponin T (cTnT) and the leukocyte count increased statistically significantly in revascularized patients at 24 and 72 h after revascularization compared to the beginning of the procedure and was always statistically significantly higher in on-pump patients. Syndecan-1 and hyaluronic acid only increased in on-pump patients 24 and 72 h after revascularization. IL-18 correlated positively with syndecan-1 and CRP only in the pump setting and with the number of leukocytes in both revascularization regimens 24 and 72 h after the surgery. cTnT and hyaluronic acid did not correlate with IL-18. Our results suggest that IL-18 plays an important role in the early inflammatory response in patients during open heart surgery and in the early postoperative period, leading to additional damage to the EG, while it is probably not responsible for myocardial necrosis. It could serve as a biomarker to identify high-risk patients and as a therapeutic target to reduce inflammation and EG degradation. In addition, measurement of IL-18 could help improve the treatment, recovery, and outcomes of patients after heart surgery.

## 1. Introduction

Cardiovascular diseases, including ischemic heart disease, are the most common cause of death in the world [1]. Ischemic heart disease is caused by a narrowing of the coronary arteries because of a complex atherosclerotic process. The formation of atherosclerotic plaques leads to a reduced blood supply to the heart tissue [2,3] and consequently to ischemia, which is eventually followed by necrosis of the myocardial cells and the development of a local inflammatory reaction and a systemic immunological response [2,3,4]. Patients with advanced ischemic heart disease, to whom medical treatment is not sufficient, require invasive cardiologic or cardiac surgical treatment. Treatment options range from percutaneous coronary intervention to open heart surgery [5,6]. Surgical myocardial revascularization is a therapeutic option when percutaneous coronary intervention is not the best option. There are two surgical techniques: one in which extracorporeal circulation is used, on-pump, and the other in which the operation is performed on the beating heart, off-pump. Both techniques have their advantages and disadvantages. On-pump surgery is performed in patients with complex multivessel disease, whereas off-pump surgery is indicated in patients with isolated anterior or limited coronary disease, in elderly patients, and in patients at high risk [7]. Although on-pump surgery has been shown to be related to long-term graft survival, a high risk of perioperative complications has been observed [8]. In addition, short-term outcomes and incidence of complications may be better in patients who have undergone off-pump surgery, but the long-term prognosis is poorer due to low graft patency and survival [9]. Unfortunately, there is still no clear distinction as to which technique is better, and the results are contradictory. The decision on which technique to use depends on the patient’s condition and the surgeon’s preference and experience. As a rule of thumb, the on-pump technique is typically used in patients with multivessel coronary artery disease or patients with multiple significant comorbidities, while the off-pump technique is beneficial in patients with certain conditions such as calcification of the ascending aorta, significant lung disease, or end-stage kidney disease [6,7,8,9,10,11,12,13].

Regardless of the technique used, ischemia–reperfusion injury (IRI) of the myocardium and endothelial dysfunction (ED) occurs after the procedure, which can cause further complications [14,15]. This leads to a strong inflammatory response and the release of inflammatory mediators, including interleukin (IL)-18, an important cytokine regulator involved in innate and adoptive immunity [16,17,18,19,20,21,22,23,24]. It is produced by various cell types, including cardiomyocytes [25], and its elevated levels can be detected after heart failure, myocardial infarction, and stroke [26,27]. In addition to the inflammatory response, IL-18 can damage the endothelial glycocalyx (EG) by other mechanisms. During the reperfusion period, a large amount of reactive oxygen species (ROS) is generated. ROS activate the MAPK and NF-kB signaling pathways, which induce proapoptotic Fas and Fas-L and lead to cell apoptosis. Furthermore, ROS promote the establishment of a positive feedback loop in mitochondria by opening the mitochondrial permeability transition pore, leading to additional ROS production, mitochondrial damage, and cell death. Furthermore, IL-18 promotes the release of mitochondrial cytochrome c and the subsequent activation of caspase-9, leading to cell apoptosis. IL-18 causes additional damage to the endothelial glycocalyx (EG) and thus promotes ED [28,29]. A damaged EG releases various products that could be potential biomarkers for patients undergoing cardiac surgery [30,31].

The EG, a dynamic gel-like layer lining the luminal surfaces of endothelial cells, plays a crucial role in vascular homeostasis, regulation of permeability, and mechanotransduction [32,33]. It consists mainly of proteoglycans, glycosaminoglycans, and associated plasma proteins. Since the EG covers the luminal surfaces of the vascular endothelial cells, it is in direct contact with the bloodstream, which generates shear stress that compromises the integrity of the EG [34]. The protein core of the EG consists of transmembrane syndecans (syndecans 1–4) and membrane-bound glypicans [35]. Syndecan-1 is the dominant form of syndecans and is involved in the promotion of inflammation, infectious diseases, and tumors [36]. The proteoglycans syndecan and glypican bind glycosaminoglycans (heparan sulfate, chondroitin sulfate, dermatan sulfate, hyaluronic acid) [34]. Thanks to their long-chain molecules, glycosaminoglycans move with the bloodstream and are the binding sites for various blood cells, receptors, and proteins [37]. Hyaluronic acid has a linear, non-sulfated structure and interacts with the receptor CD44 on the surface of vascular endothelial cells [38]. Although hyaluronic acid accounts for only 5 to 20% of total EG glycosaminoglycans, it has several important functions. It is essential for the thickness and flexibility of the EG and thus for some of its key properties such as mechanotransduction, selective permeability, and limited adhesion and penetration of leukocytes [39]. Under physiological conditions, hyaluronic acid therefore exhibits anti-inflammatory and immunosuppressive properties [40]. Studies have shown that hyaluronic acid, together with chondroitin sulfate, is the most susceptible to structural and chemical changes caused by ROS [38]. This leads to fragmentation of hyaluronic acid, which further stimulates the production of cytokines and increases the intensity of inflammation [39]. Therefore, any alteration of the EG caused by certain conditions (IRI, atherosclerosis, diabetes, sepsis) impairs its protein binding capacity [35]. The consequence of EG damage is the release (shedding) of syndecans and hyaluronic acid in the blood, which further promote the secretion of pro-inflammatory cytokines [41]. In patients undergoing cardiac surgery, early release of syndecan-1 and consequently hyaluronic acid and chondroitin sulfate bound to syndecan-1 has been observed during reperfusion [41,42]. Their early shedding during reperfusion indicates significant endothelial injury and EG degradation, which may exacerbate IRI.

IRI, which is common in cardiac surgery, leads to oxidative stress, inflammation, and ED, resulting in further disruption of the EG. The extent of myocardial damage during this process is often assessed by cTnT, a highly specific biomarker of cardiomyocyte damage. In addition, systemic inflammatory markers such as C-reactive protein (CRP) and leukocyte count serve as indicators of the inflammatory response induced by surgical stress and reperfusion injury [43]. Understanding the interplay between EG degradation, myocardial injury, and inflammation could provide valuable insight into strategies to mitigate ED and improve patient outcomes after cardiac surgery.

The aim of this study was to analyze the influence of on-pump and off-pump coronary artery bypass surgery on the concentration of IL-18 and the degradation products of the EG (syndecan-1, hyaluronic acid) in the peripheral and cardiac circulation and their correlations to ischemia–reperfusion myocardial injury during open heart surgery and in the early postoperative period.

## 2. Results

### 2.1. Demographic and Clinical Data of Patients

The demographic and clinical data of patients who underwent on-pump (on-pump group) and off-pump (off-pump group) coronary artery bypass grafting are shown in Table 1. Patients did not differ in age, gender, duration of mechanical ventilation, length of intensive care unit (ICU) stay, and outcome.

### 2.2. The Influence of On-Pump and Off-Pump Coronary Artery Bypass Surgery on the Absolute Number of Leukocytes, the Concentration of C-Reactive Protein, and Cardiac Troponin T

**Figure 1:**
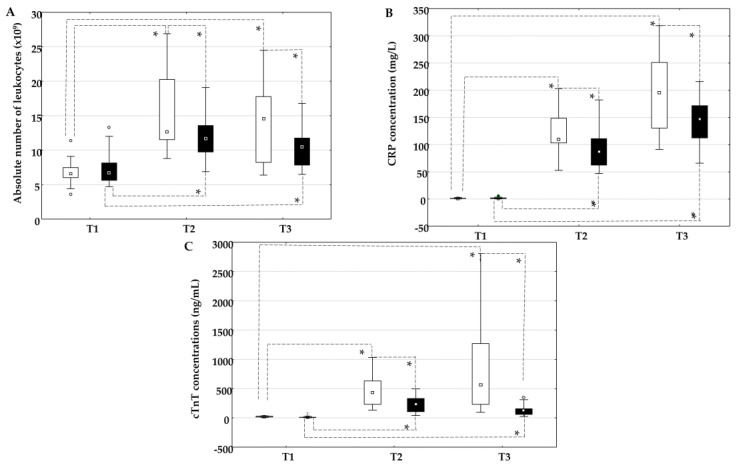
Comparison of the absolute number of leukocytes (**A**), the concentration of CRP (**B**), and the concentration of cTnT (**C**) in the on-pump group (□) and the off-pump group (■) before surgery (T1) and 24 h (T2) and 72 h (T3) after surgery in peripheral blood. * Level of statistical significance: *p* < 0.05. Data are expressed as median and 25th–75th percentile. CRP—C-reactive protein, cTnT—cardiac troponin T.

The absolute number of leukocytes (Figure 1A), the concentration of CRP (Figure 1B), and the concentration of cTnT (Figure 1C) increased statistically significantly in the on-pump group and in the off-pump group at time points T2 and T3 compared to time point T1. In addition, the absolute number of leukocytes, the concentration of CRP, and the concentration of troponin were higher in the on-pump group than in the off-pump group at time points T2 and T3.

### 2.3. The Influence of On-Pump and Off-Pump Coronary Artery Bypass Surgery on the Concentration of IL-18

**Figure 2:**
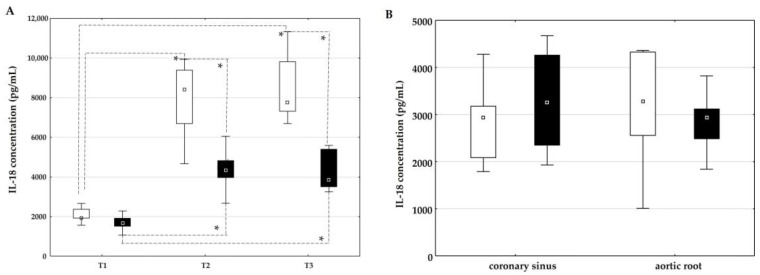
Dynamic changes in and comparison of the concentration of interleukin-18 between the on-pump group (□) and the off-pump group (■) before surgery (T1) and 24 h (T2) and 72 h (T3) after surgery in peripheral blood (**A**) and in the coronary sinus and aortic root during surgery before bypass grafting was performed (**B**). * Level of statistical significance: *p* < 0.05. Data are expressed as median and 25th–75th percentile.

The influence of on-pump and off-pump coronary artery bypass surgery on the concentration of IL-18 is shown in Figure 2. The plasma concentration of IL-18 was statistically significantly higher at time points T2 and T3 compared to time point T1 in both groups (Figure 2A). In addition, a statistically higher plasma concentration of IL-18 was observed in the on-pump group than in the off-pump group at time points T2 and T3 (Figure 2A).

The concentration of IL-18 in the coronary sinus and aortic root did not differ between the groups (Figure 2B).

### 2.4. The Influence of On-Pump and Off-Pump Coronary Artery Bypass Surgery on Endothelial Glycocalyx Degradation Products

**Figure 3:**
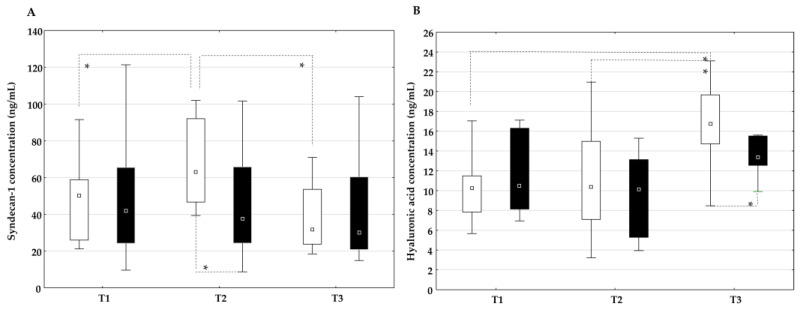
Dynamic changes in and comparison of the plasma concentration of syndecan-1 (**A**) and hyaluronic acid (**B**) between the on-pump group (□) and the off-pump group (■) at different time points: before surgery (T1) and 24 h (T2) and 72 h (T3) after surgery. * Level of statistical significance: *p* < 0.05. Data are expressed as median and 25th–75th percentile.

In the on-pump group, the plasma concentration of syndecan-1 increased significantly at time T2 compared to time T1 and decreased at time T3, while the plasma concentration of syndecan-1 in the off-pump group did not change significantly at all time points (Figure 3A). In addition, a statistically higher plasma concentration of syndecan-1 was observed at time point T2 in the on-pump group than in the off-pump group. The plasma concentration of hyaluronic acid increased significantly at time T3 in the on-pump group but not in the off-pump group (Figure 3B). In addition, a higher plasma concentration of hyaluronic acid was observed at time T3 in the on-pump group than in the off-pump group.

**Figure 4:**
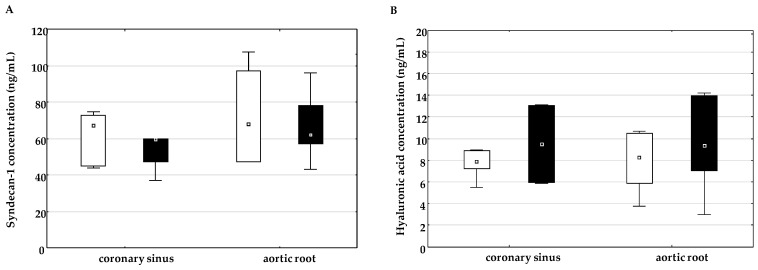
Comparison of the concentration of syndecan-1 (**A**) and hyaluronic acid (**B**) in the coronary sinus and aortic root between the on-pump group (□) and the off-pump group (■) during surgery before bypass grafting was performed. Data are expressed as median and 25th–75th percentile.

The concentration of syndecan-1 (Figure 4A) and hyaluronic acid (Figure 4B) in the coronary sinus and aortic root did not differ between the groups.

### 2.5. Correlation Between the Plasma Concentration of IL-18 and Markers of the Inflammatory Response, Myocardial Injury, and Endothelial Glycocalyx Degradation Products

The correlations between the concentration of IL-18 and the absolute number of leukocytes, the concentration of CRP, troponin, syndecan-1, and hyaluronic acid are shown in Table 2. A positive correlation was observed between the concentration of IL-18 and the absolute number of leukocytes, between the concentration of IL-18 and the concentration of CRP, and between the concentration of IL-18 and the concentration of syndecan-1 at time points T2 and T3 in the on-pump group. In the off-pump group, only a positive correlation between the concentration of IL-18 and the concentration of the absolute number of leukocytes was found at time points T2 and T3.

## 3. Discussion

### 3.1. Cardiac Surgery and IL-18

Cardiac surgery, particularly when performed with a cardiopulmonary bypass (on-pump), presents a significant physiological challenge due to the associated systemic inflammatory response and IRI [44]. One of the critical elements affected during cardiac surgery is the EG, damage to which contributes to ED, increased vascular permeability, and the subsequent cascade of inflammatory responses [45]. In these processes, IL-18, a member of the proinflammatory interleukin family that is elevated in patients with cardiovascular disease, has been shown to be a potential key factor in postoperative inflammatory and ischemic responses [45,46]. It has been shown that an increased concentration of circulating IL-18 is closely related to inflammation, subsequent IRI injury, and poor prognosis in patients undergoing cardiac surgery. Neutralization of IL-18 by anti-IL-18 blocking antibody or IL-18 binding protein effectively ameliorated IRI-induced myocardial injury [47]. However, the role of IL-18 in these processes is not entirely elucidated and needs further scientific attention. Therefore, our aim was to investigate the role of IL-18 in patients that underwent open heart surgery and in the early postoperative period and its impact on endothelial integrity.

### 3.2. Diagnostic, Prognostic, and Potential Therapeutic Implications of Studied Biomarkers

Our results showed significantly increased IL-18 concentrations in the peripheral blood in both groups of patients, suggesting that these increases can be influenced not only by CPB but also by surgical trauma, surgical techniques, myocardial manipulations, ischemia, or pre-existing inflammation in patients. This increase in IL-18 concentration followed a similar pattern to general inflammatory markers, such as the number of leukocytes and CRP. Concentrations of IL-18 and CRP and the number of leukocytes were consistently higher in patients who underwent on-pump surgery, indicating greater systemic inflammation in these patients compared to patients without CPB, which is consistent with generally accepted findings [48]. Accordingly, IL-18 and CRP correlated positively only in the on-pump group 24 and 72 h after surgery, although IL-18 and leukocyte count were positively correlated in both groups. In addition, IL-18 is closely related to myocardial IRI and poor prognosis of patients with ischemic heart disease [49]. An increased concentration of IL-18 in the peripheral circulation has been associated with higher risk and incidence of ischemic events [50], including myocardial infarction [51]. Consistent with these findings, cTnT concentrations increased in our patients after revascularization. In a mouse model, modulation of IL-18 activity by administration of anti-IL-18 antibodies or mesenchymal stem cells overexpressing IL-18 binding protein has been shown to reduce myocardial infarct size [52,53]. In our study, IL-18 and cTnT did not correlate 24 h or 72 h after revascularization surgery in either patient group, suggesting that the revascularization process itself and not the systemic inflammation induced by IL-18 is responsible for the early troponin increase. Prognostically significant myocardial damage, as evidenced by an elevated cTnT concentration [54], is one of the most common complications after cardiac surgery and is associated with increased mortality [55,56].

On the other hand, the greater increase in cTnT levels in the on-pump group underscores the greater extent of myocardial IRI associated with extracorporeal circulation, which is consistent with generally accepted findings [57].

### 3.3. Cardiac Surgery and Endothelial Glycocalyx

One of the critical elements affected during cardiac surgery is the EG, damage to which contributes to ED, increased vascular permeability, and the subsequent cascade of inflammatory responses [58]. In these processes, IL-18, a member of the proinflammatory IL-1 family that is elevated in patients with cardiovascular disease, has been shown to be a potential key factor in postoperative inflammatory and chronic ischemic responses [49]. As a proinflammatory cytokine, IL-18 contributes to EG degradation. Namely, proinflammatory cytokines released during cardiac surgery activate various proteases, including matrix metalloproteinases (MMPs), which cleave the syndecan ectodomain, and hyaluronidase, which degrades hyaluronic acid [59]. In our study, we did not find significant differences in the concentrations of IL-18 and the EG degradation products syndecan-1 and hyaluronic acid in the coronary sinus and aortic root blood samples in the on-pump and off-pump groups immediately before the revascularization process, in contrast to those after revascularization. This suggests that revascularization increases systemic inflammation mediated by IL-18 and EG damage, particularly in on-pump patients, as IL-18 correlates positively with syndecan-1 and CRP concentrations only in this group of patients and correlates with lymphocytes in both groups. Hyaluronic acid did not correlate with IL-18 [60]. A possible reason for this difference in the dynamics of changes in the concentrations of EG degradation products could be that syndecan-1 is more susceptible to inflammatory mediators induced by surgical techniques and the effects of extracorporeal circulation, and it therefore had a higher concentration in the period closer to surgery (T2), while the concentration decreased in the next period (T3). This result is consistent with the known effects of extracorporeal circulation, which include non-physiological blood flow, hemodilution, mechanical shear stress, and exposure to artificial surfaces, all of which can contribute to EC degradation, inflammation, and ROS formation [28]. On the other hand, the dynamics of hyaluronic acid suggest that it may be caused by oxidative damage. It is known that hyaluronic acid is one of the EG components most sensitive to ROS [31]. ROS and reactive nitrogen species (RNS) can cause not only intraoperative but also postoperative damage, which is consistent with the continuous increase in hyaluronic acid concentration. It may also lead to atrial fibrillation and myocardial ischemia [60], which is consistent with the statistically significant gradual increase in troponin levels in both surgical procedures (on- and off-pump), although patients who underwent on-pump surgery had higher troponin concentrations [53]. The positive correlation of CRP concentration with IL-18 in the on-pump group but not in the off-pump group could be a result of different inflammatory pathways activated with those two procedures. Namely, the pulsatile nature of the on-pump procedure causes rapid production of ROS, which promotes inflammatory events and a rise in CRP [28]. On the other hand, the slower development of inflammation in the off-pump procedure could be more related to surgical trauma and stress. In this case, inflammation in blood vessels develops through the activation of lymphocytes and mononuclear cells, which produce and release pro-inflammatory cytokines, mostly IL-6 and TNF-α. TNF-α further induces IL-6 production, while IL-6 stimulates the production of CRP in the liver [61].

### 3.4. Study Limitations

This study has several limitations. First, the number of time points analyzed was limited, which may have missed important dynamics in the inflammatory and endothelial response. More frequent sampling, particularly in the first few hours after surgery, would provide a more detailed temporal profile. Furthermore, measuring the concentration of IL-18 and the degradation products of EG after 72 h could be useful to gain additional insight into the resolution phase of inflammation and the recovery of the EG. Second, the sample size was relatively small, limiting the statistical power to detect subtle but clinically important differences. Third, the study focused primarily on a limited number of biomarkers. The inclusion of additional inflammatory mediators such as IL-6 and TNF alpha as well as endothelial markers would therefore enable a more comprehensive understanding of the investigated processes and provide deeper insights into the mechanisms of endothelial damage in cardiac surgery.

### 3.5. Future Perspectives of IL-18 in Cardiac Surgery

Given the significant association of IL-18 with inflammatory markers and IRI, further research should focus on evaluating its prognostic potential in larger patient cohorts. Long-term studies tracking IL-18 concentrations and patient outcomes may reveal whether IL-18 is a useful marker for predicting postoperative risk. IL-18 could potentially serve as a predictive biomarker for postoperative ED. While the study suggests that IL-18 is not directly involved in myocardial necrosis, its strong association with early inflammation and ED suggests that it could help identify patients at risk for vascular complications after cardiac surgery. Elevated IL-18 levels in the early postoperative period could indicate an increased inflammatory state and impending ED.

In addition, investigations of the ED using non-invasive techniques and soluble endothelial markers could provide further insights into the vascular consequences of EG damage. Therapeutic strategies targeting IL-18 in the context of cardiac surgery should be further analyzed and may offer new therapeutic options. Extending its clinical utility, IL-18 measurement could be integrated into risk stratification protocols to perform intensive surveillance in high-risk patients.

**Figure 5:**
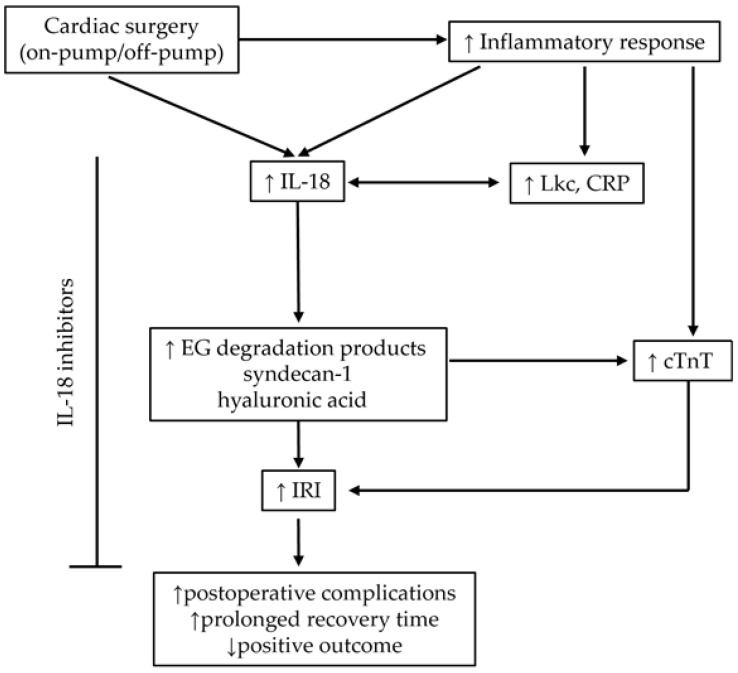
Summary of the study results with clinical implications. Cardiac surgery leads to an increased inflammatory response that triggers degradation of EG. A more intense inflammatory response leads to higher EG degradation, which in turn triggers IRI. IRI increases postoperative complications and prolongs the recovery time and positive outcome. CRP—C-reactive protein, cTnT—cardiac troponin, EG—endothelial glycocalyx, IL—interleukin, Lkc—leukocytes, IRI—ischemia–reperfusion injury. ↑, increase; ↓, decrease.

A summary of the study results and their implications on clinical outcomes is summarized in Figure 5.

## 4. Materials and Methods

### 4.1. Patients

This prospective study included patients scheduled for elective coronary artery bypass surgery between May 2023 and December 2024 at the Department of Anesthesiology, Intensive Care and Pain Medicine, the Department of Cardiac Anesthesia and the Department of Cardiac Surgery at the University Hospital Rijeka, Rijeka, Croatia. The protocol was approved by the Ethics Committee of the Clinical Hospital Centre Rijeka and the Committee of the Faculty of Medicine Rijeka, University of Rijeka, Rijeka, Croatia, and was carried out according to the World Medical Association criteria in the Declaration of Helsinki. All patients received an information form and an additional explanation from the research staff. Patients were enrolled in the study after signing an informed consent form. The decision to perform cardiac bypass surgery was based on the guidelines of the European Society of Cardiology (ESC) and the European Association for Cardio-Thoracic Surgery (EACTS) [62].

**Figure 6:**
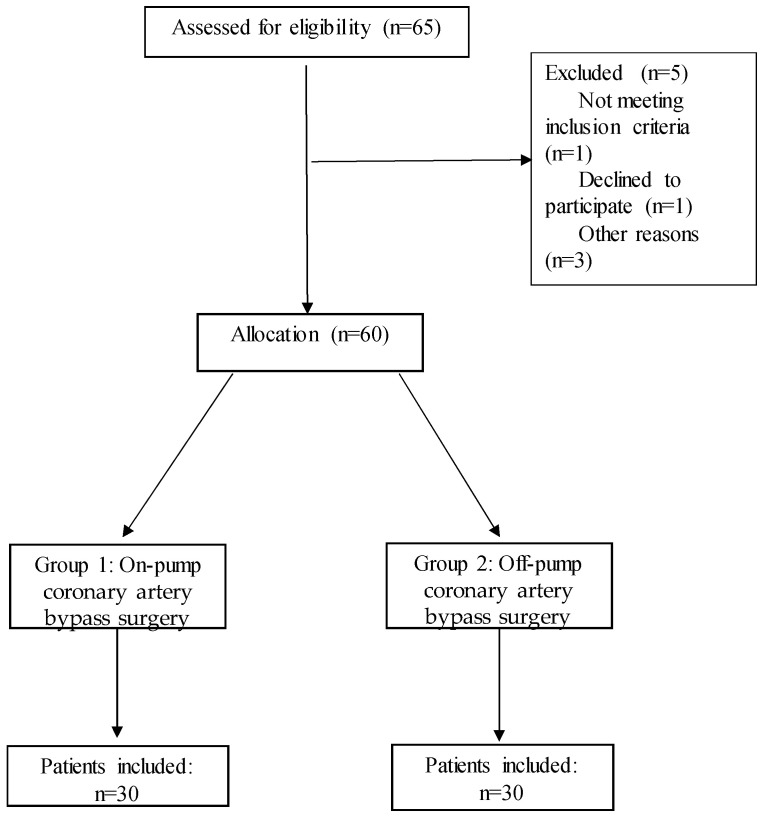
Flow diagram of the study protocol.

The study included 60 patients who had undergone surgical myocardial revascularization. The patients were divided into two groups according to the surgical technique used. The on-pump group consisted of patients who underwent surgery using the on-pump technique, while the off-pump group consisted of patients who underwent surgery using the off-pump technique (Figure 6). The choice of technique depended on the surgeon and was in accordance with the 2021 ACCF/AHA guidelines [13]. Surgeons were aware of the ongoing study but were blinded when the patients were enrolled in the study until the decision on the surgical technique was made to minimize potential bias in the choice of technique.

Patients younger than 18 and older than 80 years with confirmed autoimmune disease, malignant disease, patients taking corticosteroid or immunosuppressive therapy, and patients with a history of coagulopathy were excluded from the study.

Demographic data, including sex and age, as well as duration of surgery and anesthesia, duration of mechanical ventilation and ICU stay, and outcome were recorded.

### 4.2. Anesthesia and Surgical Procedure

Upon arrival in the operating room, the patients were preoxygenated. General anesthesia was induced with intravenous administration of the sedative propofol (Propofol-Lipuro, B. Braun Melsungen AG, Melsungen, Germany) at 1.5–2 mg/kg and sufentanil at 0.4 μg/kg (Sufentanil Altamedics, Laboratoire Renaudin, Itxassou, France) followed by a bolus of 0.6 mg/kg rocuronium (Rocuronium bromide, B. Braun Melsungen AG, Melsungen, Germany). All patients were endotracheally intubated and mechanically ventilated with protective settings while maintaining normocapnia. Anesthesia was maintained by additional doses of sufentanil and rocuronium bromide as well as sevoflurane (Sevofluran Baxter, Baxter S.A., Lessines, Belgium) with a MAC of 0.7–1.5. FiO_2_ was titrated to an SpO_2_ between 94–98%. Vasoactive drugs, tranexamic acid, heparin, protamine, transfusions, and hemostatic agents were administered according to the hospital protocol and the attending anesthesiologist.

The surgical procedure was performed through a median sternotomy. Cardiopulmonary bypass surgery was performed with an Affinity Fusion Oxygenator Filter (Medtronic, Minneapolis, MN, USA). Mean arterial pressure was maintained between 50 and 80 mmHg, and blood flow was maintained at 2.4 L/min per m^2^. Mild systemic hypothermia was applied. Cardioplegia with Calafiore blood was administered via retrograde cardioplegic cannula to preserve the heart. Off-pump cardiopulmonary bypass surgery procedures were performed on the beating heart using the Octopus IV system (Medtronic Inc., Minneapolis, MN, USA). Regardless of the type of revascularization, epicardial leads of a temporary pacemaker were placed in all patients, as well as two mediastinal chest drains, and one thoracic chest drain was used if the pleural cavity was opened. After surgery, all patients were transferred to the intensive care unit (ICU) for postoperative care. After admission to the ICU, we aimed for early weaning and extubation if the criteria were met.

### 4.3. Blood Collection

Five milliliters of peripheral venous blood and two milliliters of blood from the coronary sinus and aortic root were collected in heparin vacutainer blood collection tubes (Becton Dickinson, Erembodegem, Belgium) from each patient participating in the study. Peripheral venous blood was collected before surgery (T1) and 24 (T2) and 72 h (T3) after surgery, while blood from the coronary sinus and aortic root was collected during surgery before bypass grafting was performed.

Plasma was collected by centrifugation at 200× *g* for 10 min and stored at −80 °C until further analysis.

### 4.4. Determination of the Absolute Number of Leukocytes, the Concentration of C-Reactive Protein, and the Concentration of Troponin

The absolute number of leukocytes was determined using an electronic counter (Technicom H-1 System; Technicon Instrument Corporation, New York, NY, USA) according to the laser-optical principle of dark colors. The concentration of CRP and cTnT was determined using an automatic analyzer (Olympus, Tokyo, Japan).

### 4.5. Determination of the Plasma Concentration of Interleukin-18, Syndecan-1, and Hyaluronic Acid

Plasma concentrations of IL-18, syndecan-1, and hyaluronic acid were quantified using high-sensitivity enzyme-linked immunosorbent assays (ELISAs) obtained from MyBioSource (San Diego, CA, USA) following the manufacturer’s protocols. Briefly, plasma samples and standards were added to microplate wells pre-coated with specific antibodies. After incubation and washing to remove unbound substances, a biotin-conjugated detection antibody was added, followed by streptavidin–horseradish peroxidase. The colorimetric reaction was developed using TMB substrate and then stopped with the provided acidic solution. Optical density was measured using a microplate reader (EL808, BioTek Instruments, Winooski, VT, USA) at a primary wavelength of 450 nm with a reference wavelength of 630 nm. Concentrations were calculated based on standard curves generated for each analyte and expressed in pg/mL. Data were analyzed using online analysis software (https://www.myassays.com/, Brighton, East Sussex, UK).

### 4.6. Statistical Analysis

Statistical analyses were performed using data analysis software (Statistica 14.0.0.; TIBCO Software Inc., Palo Alto, CA, USA). The normality of the distribution was checked using the Kolmogorov–Smirnov test. If the data did not show a normal distribution, a non-parametric statistical analysis was performed. Friedman’s test and the post hoc Wilcoxon rank sum test were used to compare time points within groups. The Kruskal–Wallis non-parametric test was used to analyze differences between groups, followed by the Mann–Whitney U-test for post hoc analysis. Bonferroni’s adjustments were applied when multiple comparisons were made. The Mann–Whitney U-test was used to analyze the differences between the groups. The sample size was calculated based on our preliminary results for the groups. We set the type I error rate (alpha) to 0.05, the discriminatory power to 0.80, and 26 patients in each group were required. The correlation analysis was performed using the Spearman rank correlation coefficient. Categorical variables were analyzed using the chi-square test (χ^2^) or Fisher’s exact test, as appropriate. The difference was considered statistically significant at a *p* value < 0.05. All data are presented as 25th–75th percentile values.

## 5. Conclusions

Our results confirm that open heart surgery leads to a pronounced systemic inflammatory response and degradation of the EG in the early postoperative period. Our study emphasizes the important role of IL-18 in the inflammatory response, ED, and the ischemic myocardial injury that occurs shortly after surgery, particularly in patients who have undergone on-pump coronary artery bypass grafting surgery. Since IL-18 is associated with inflammation, ED, and IRI, it could be useful in two ways: first, as a biomarker to identify patients at higher risk for complications, and second, as a potential target for therapy to reduce inflammation and ED and protect the heart during and after high-risk procedures such as open heart surgery. Therefore, measuring IL-18 could provide important insights into how patients respond to cardiac surgery and could lead to new strategies to improve patient recovery and clinical outcomes.

## Figures and Tables

**Table 1:** Basic demographic and clinical data of the patients.

	On-Pump Group (n = 30)	Off-Pump Group (n = 30)	*p* Value
Age (years)	68.4 (59–74.5)	67 (63–69)	0.47
Sex (men/women)	19/11	18/12	0.39
Length of mechanical ventilation (hours)	10 (9–13.1)	10 (9–11)	0.14
Length of intensive care unit stay (hours)	24 (24–48)	24 (24–36)	0.71
Outcome (discharged/death)	30/0	30/0	1

Continuous variables are presented as median and 25th–75th percentiles, and categorical data are presented as number of cases (n). On-pump group represents patients who underwent on-pump coronary artery bypass surgery, and off-pump group represents patients who underwent off-pump coronary artery bypass surgery.

**Table 2:** Spearman correlation between the concentration of IL-18, absolute number of leukocytes, the concentration of CRP, troponin, syndecan-1, and hyaluronic acid in the on-pump group and the off-pump group at different time points: before surgery (T1) and 24 h (T2) and 72 h (T3) after surgery.

Correlation	Concentration of IL-18 (pg/mL)											
	On-Pump Group						Off-Pump Group					
	T1		T2		T3		T1		T2		T3	
	r	*p*	r	*p*	r	*p*	r	*p*	r	*p*	r	*p*
Absolute number of leukocytes (×10^9^)	0.70	0.18	0.74	0.013	0.83	0.04	0.18	0.61	0.71	0.019	0.41	0.02
C-reactive protein concentration (mg/L)	0.74	0.15	0.73	0.016	0.84	0.03	0.16	0.67	0.49	0.15	0.33	0.38
Cardiac troponin T concentration (ng/mL)	0.40	0.59	0.89	0.41	0.71	0.15	0.07	0.84	0.19	0.06	0.64	0.16
Syndecan-1 concentration (ng/mL)	0.57	0.31	0.82	0.022	0.73	0.01	0.37	0.31	0.42	0.22	0.05	0.89
Hyaluronic acid concentration (ng/mL)	0.19	0.81	0.25	0.45	0.32	0.52	0.21	0.61	0.12	0.72	0.25	0.51

*p*—level of statistical significance, r—correlation coefficient.

## Data Availability

Data is available upon request.

## Abbreviations

The following abbreviations are used in this manuscript:

CABG	Coronary artery bypass grafting
CRP	C-reactive protein
ED	Endothelial dysfunction
EG	Endothelial glycocalyx
ICU	Intensive care unit
IL	Interleukin
IRI	Ischemia–reperfusion injury
